# Expression Proteomics and Histone Analysis Reveal Extensive Chromatin Network Changes and a Role for Histone Tail Trimming during Cellular Differentiation

**DOI:** 10.3390/biom14070747

**Published:** 2024-06-24

**Authors:** Giorgio Oliviero, Kieran Wynne, Darrell Andrews, John Crean, Walter Kolch, Gerard Cagney

**Affiliations:** 1 Systems Biology Ireland, School of Medicine, University College Dublin, D04 V1W8 Dublin, Ireland; kieran.wynne1@ucd.ie (K.W.); walter.kolch@ucd.ie (W.K.); 2 Conway Institute of Biomolecular & Biomedical Research, University College Dublin, D04 V1W8 Dublin, Ireland; darrell.andrews@ucd.ie (D.A.); john.crean@ucd.ie (J.C.); 3 School of Biomolecular & Biomedical Research, University College Dublin, D04 V1W8 Dublin, Ireland

**Keywords:** epigenetic, histone modification, differentiation, expression proteomics, protein interaction

## Abstract

In order to understand the coordinated proteome changes associated with differentiation of a cultured cell pluripotency model, protein expression changes induced by treatment of NT2 embryonal carcinoma cells with retinoic acid were monitored by mass spectrometry. The relative levels of over 5000 proteins were mapped across distinct cell fractions. Analysis of the chromatin fraction revealed major abundance changes among chromatin proteins and epigenetic pathways between the pluripotent and differentiated states. Protein complexes associated with epigenetic regulation of gene expression, chromatin remodelling (e.g., SWI/SNF, NuRD) and histone-modifying enzymes (e.g., Polycomb, MLL) were found to be extensively regulated. We therefore investigated histone modifications before and after differentiation, observing changes in the global levels of lysine acetylation and methylation across the four canonical histone protein families, as well as among variant histones. We identified the set of proteins with affinity to peptides housing the histone marks H3K4me3 and H3K27me3, and found increased levels of chromatin-associated histone H3 tail trimming following differentiation that correlated with increased expression levels of cathepsin proteases. We further found that inhibition of cathepsins B and D reduces histone H3 clipping. Overall, the work reveals a global reorganization of the cell proteome congruent with differentiation, highlighting the key role of multiple epigenetic pathways, and demonstrating a direct link between cathepsin B and D activity and histone modification.

## 1. Introduction

Cellular differentiation programmes, such as differentiation into specialized cells from the pluripotent state, involve coordinated changes to the expression levels and function of thousands of proteins. While it has been challenging to understand these processes at a global level, recent analyses have emphasized regulation by chromatin-based expression systems, and in particular extensive use of dynamic write–read–rewrite control of histone modifications including acetylation and methylation [1]. Mass spectrometry uniquely has the ability to analyse proteins on this scale, and can approach comprehensive coverage, particularly if the proteome is simplified by fractionation. In the ‘bottom-up’ mass spectrometry approach, peptides arising from trypsin digestion of protein lysates are separated by HPLC and identified on the basis of their tandem MS2 spectra, while the parent MS1 ion signals can be used to determine the relative abundance of the parent protein using the so called ‘label-free quantitation’ approach [2]. Fruthermore Scientific, Irelandre, the resulting mass spectral datasets also include descriptions of the state of many protein post-translational modifications (PTMs), providing information on an additional layer of potential protein regulation.

In order to investigate global proteome rearrangements during a cellular differentiation programme from a pluripotent state, we used high resolution/high mass accuracy mass spectrometry, timsTOF Pro instrument (Bruker Daltonics; Bremen, Germany) with the PASEF acquisition mode. NTERA2 (NT2) cells were stimulated with retinoic acid to induce differentiation. This cell line is derived from a metastatic embryonal carcinoma and displays properties typical of pluripotent cells, for example expression of the OCT4 and NANOG transcription factors. Cultured NT2 cells are widely used as an experimental model of differentiation [3,4], developing neuron-like properties upon treatment with retinoic acid [5,6].

Retinoic acid, derived from vitamin A (retinol), is present in vertebrates in various stereoisomeric forms. The all-*trans* form (ATRA) predominates and plays the greatest role during development [7]. Retinoic acid initiates signalling events that relay gene expression instructions in the nucleus. This form of regulation is particularly important in events such as embryogenesis and the development of tissues and organs. Fruthermore Scientific, Irelandre, the resulting transcription programmes, involving alternative activation and repression of genes that promote and inhibit differentiation and pluripotency factors, respectively, rely critically on epigenetic regulatory pathways [8].

Epigenetic pathways exploit a wide variety of mechanisms, including nucleosome remodelling, DNA and histone modification, miRNA regulation, and the activation of DNA elements such as enhancers [9]. For example, the Polycomb Repressor Complexes PRC1 (an E3 ubiquitin ligase) and PRC2 (a histone methyltransferase) cooperate to generate repressed chromatin domains that may be >100 kilobases long and form long range 3D topological structures [10]. Polycomb domains are enriched in the Polycomb proteins themselves and are characterized by the presence of specific histone marks (H2AK119ub, H3K27me3). Other heterooligomeric protein complexes can generate histone marks associated with the activation or repression of gene expression (e.g., histone acetyltransferases and deacetylases, histone methyltransferases and demethylases) [11] or the remodelling of chromatin by manipulation of nucleosomes to facilitate access to the gene expression machinery (e.g., SWI/SNF) [12].

There is abundant evidence linking epigenetic pathways to extended differentiation programmes. For example, PRC1 regulates the expression of developmental marker genes including Meis1/2 [13], while PRC2 collaborates with the nuclear corepressors NCOR1 and NCOR2 in a retinoic acid-dependent manner to effect repression of the Fgf8 locus during mouse body axis development [14]. Other mechanisms by which epigenetic processes regulate differentiation pathways include the activation of transcriptional programmes via mitogen activated kinase (MAPK) signalling [15,16], the integration of molecular instructions arising from interplay among diverse DNA elements such as promoters, enhancers, and silencers [17], chromatin remodelling via signalling by transcription factors [18], and the recruitment of co-activator and co-repressor proteins such as the SMAD transcription factors [19].

Here, we investigated a cultured cell model of differentiation, focusing on an attempt to identify key protein abundance and functional change that may underlie the observed phenotype. We find widespread changes in the proteome, particularly among chromatin proteins regulating and interacting with epigenetic pathways. We therefore focused on quantifying the relative expression of histone marks over the differentiation cycle, as well as using histone peptide pulldown experiments to identify proteins with binding affinity to the H3K4me3 and H3K27me3 marks (associated with gene activation and repression, respectively). Finally, we report a link between proteolysis of histone H3 (‘clipping’) and the proteins cathepsin B and Cathepsin D.

## 2. Materials and Methods

### 2.1. Cell Culture and Differentiation of NTERA Cells

NTera-2/cloneD1 (NT2) cells (ATCC, CRL-1973) were cultured in 92 mm tissue culture dishes (Nunclon, Fisher Scientific, Dublin, Ireland) in Dulbecco’s Modified Eagle Medium (DMEM) supplemented with 10% (*v*/*v*) fetal bovine serum (Hyclone, Logan, UT, USA), 100 U/mL penicillin and 100 U/mL streptomycin (Gibco, Fisher Scientific, Dublin, Ireland). Cells were passaged by trypsinizing with 0.25% Trypsin-EDTA (Invitrogen, Fisher Scientific, Dublin, Ireland) and plated at a ratio of 1:6. Induced neuronal differentiation was performed as described before [20]. To induce neuronal differentiation, 10 μM all-*trans* retinoic acid (RA) (Merck, Cork, Ireland) was added to the media once cells reached a density of ~50%. During the 8-day differentiation time course, the media was changed every 2–3 days. HEK293T cells were grown in DMEM medium supplemented with 10% (*v*/*v*) FBS (Hyclone, Fisher Scientific, Dublin, Ireland), 100 U/mL penicillin, and 100 U/mL streptomycin (Gibco, Waltham, MA, USA).

### 2.2. Real-Time Quantitative PCR

Extracted RNA was used to generate cDNA by reverse transcriptase PCR using the TaqMan Reverse Transcription Kit (Applied Biosystems Fisher Scientific, Dublin, Ireland). Relative mRNA expression levels were determined using the SYBR Green I detection chemistry on a LightCycler 480 II Real-Time PCR System (Fisher Scientific, Dublin, Ireland). Expression of the ribosomal gene RPO was used for normalisation. The primers used are listed in the Supplementary Materials.

### 2.3. Cell Viability Assay

NT2 cells were subjected to crystal violet staining using 0.1% crystal violet in 12-well plates (Merck, Rahway, NJ, USA). After staining, the wells were washed with phosphate-buffered saline (pH = 7.4) to remove unbound crystal violet and residual NT2 cells. The plates were then air-dried at room temperature, and 95% ethanol was added to the wells to resuspend the adhered stained cells. The ethanol-bound crystal violet stains of adhered cells were quantified by measuring at 590 nm on a spectrophotometer.

### 2.4. Immunoblotting

The immunoblotting assay was performed as described [20]. Protein lysate was quantified using the Bradford assay (Fisher Scientific, Dublin, Ireland), separated on SDS–PAGE, and transferred to nitrocellulose membranes. Membranes were blocked with 5% non-fat milk or 5% BSA at room temperature for 1 h and incubated overnight with a diluted primary antibody at 4 °C. Membranes were then washed and incubated with an HRP-conjugated goat anti-rabbit or mouse IgG secondary antibody for 1 h at room temperature. The membrane was incubated with enhanced chemiluminescence reagents (Fisher Scientific, Ireland Scientific, Dublin, Ireland) followed by exposure to X-ray films. Immunoblotting was performed using the antibodies and conditions listed in the Supplementary Materials.

### 2.5. Cell Fractionation

Whole cell lysate: NT2 cell pellets were washed in PBS and resuspended in whole cell lysis (WCL) (25 mM Tris·HCl pH 7.6, 150 mM NaCl, 1% NP40, 1% sodium deoxycholate, 0.1% SDS, 0.5 mM DTT, cOmplete Protease Inhibitor w/o EDTA (Fisher Scientific, Dublin, Ireland)). The lysates were incubated for 15 min on ice, and cell membranes were disrupted mechanically by syringing five times with a 21G narrow-gauge needle and sonicating. Lysates were incubated on ice for another 15 min and cleared by centrifugation at max speed for 15 min at 4 °C. Samples were stored at −80 °C until further use.

Cytoplasmatic and Nucleoplasmatic fractions: NT2 cell pellets were washed in PBS and resuspended in Buffer A (10 mM HEPES pH 7.9, 1.5 mM MgCl_2_, 10 mM KCl, 0.5 mM DTT, cOmplete Protease Inhibitor w/o EDTA (Fisher Scientific, Dublin, Ireland)). The lysates were incubated on ice for 30 min and cell membranes disrupted mechanically by Dounce homogenizer (pestle “A” or “loose”). Lysates were pre-cleared by centrifugation at 500× *g* for 10 min at 4 °C. Supernatants containing the “cytosol fractions” were snap frozen until further use. The remaining pellets containing intact nuclei were resuspended in Buffer B (10 mM Tris-HCl pH 7.4, 2 mM MgCl_2_, 5 mM CaCl_2_, cOmplete Protease Inhibitor w/o EDTA (Fisher Scientific, Dublin, Ireland)). The lysates were incubated for 5 min at 37 °C, then digested with MNase (1 U/μL) for 30 min at 37 °C, with gentle rocking. The digested lysates were centrifuged at 500× *g* for 10 min at 4 °C, and the supernatants containing the “Nucleoplasmatic fractions” were snap frozen until further use.

Chromatin and Histone Fractions: The remaining pellets containing chromatin and chromatin-bound material were resuspended in Buffer C (10 mM Tris-HCl pH 7.4, 2 mM MgCl_2_, 2 mM EGTA (pH ~7.0–8.0), 1 mM EDTA pH 10.0, 600 mM NaCl, 0.5 mM DTT, 5 M Sodium butyrate, 1X Deacetylase Inhibitors (Active Motif, Carlsbad, CA, USA), and 200 mM AEBSF, cOmplete Protease Inhibitor w/o EDTA (Fisher Scientific, Dublin, Ireland)) and incubated for 1 h rotating at 4 °C, in the presence of 250 U/mL benzonase nuclease. Lysates were centrifuged at 20,000× *g* for 20 min at 4 °C, and the supernatants containing the “chromatin fraction” were snap frozen until further use. Histones were purified from the remaining insoluble pellet as described before [20] with small modifications. Briefly, samples were acid extracted with 0.2 M H_2_SO_4_ for 4 h with rotation at 4 °C. The extracts were centrifuged at 3400× *g* at 4 °C for 5 min, and the supernatants were retained. Next, the supernatants were precipitated by adding 100% trichloroacetic (TCA) acid in a 1:3 ratio (*v*/*v*) overnight at 4 °C with rotation. Pellets were washed with acetone to remove residual debris and stored at −80 °C until further use.

### 2.6. Sample Preparation for Mass Spectrometry

The protein concentration of each cell fractionation was determined using the Pierce 660-nm Protein Assay (Fisher Scientific, Dublin, Ireland). A total of 20 µg of protein lysates were homogenized and denatured in urea (final concentration, 4 M), ammonium bicarbonate (100 mM), and calcium chloride (100 mM), then reduced with DTT (final concentration, 1 mM) for 15 min at room temperature and alkalinized with iodoacetamide (IAA) (3 mM) in the dark at room temperature for 15 min. The tryptic digestion protocol was performed using the automated KingFisher DuoPrime purification system (Fisher Scientific, Dublin, Ireland). Briefly, magnetic hydrophobic and hydrophilic beads were washed several times in MS-grade water and added to the deepwell plate in the KingFisher along with the samples and an equal volume of 100% ethanol. Next, the solutions were mixed at low speed for 10 min, after which the beads coupled to the proteins were collected with the magnetic arm of the KingFisher and transferred to be washed in 3 different deepwells containing 80% ethanol. The washed beads with their bound proteins were then transferred into trypsin (Promega, Madison, WI, USA, V5111)-containing deepwells at a 50:1 (*w*/*w*) protein-to-protease ratio and mixed at low speed for 8 h of digestion into peptide fragments at 37 °C. Peptide samples were transferred into low protein binding tubes, 1% of trifluoroacetic acid (TFA) was added to acidify the samples ready to be desalted, cleaned, and concentrated on C18 tips according to the manufacturer’s instructions. Purified peptides were dried in a SpeedVac. Samples were stored at −80 °C until further use. Prior to LCMS/MS analysis, samples were resuspended in low protein-binding tubes in 30 uL of 0.15% TFA acid and 1% acetic acid in mass spectrometry-grade water. Samples were stored at −80 °C until further use.

### 2.7. Mass Spectrometry Analysis

Samples were analysed on a Bruker timsTof Pro mass spectrometer (Bruker Daltonik, Bremen, Germany) connected to a Bruker nanoElute nano-lc chromatography system (Bruker Daltonik). Tryptic peptides were resuspended in water with 0.1% (*v*/*v*) trifluoroacetic acid and spiked with Hi3 peptide standards (Waters, County Wexford, Ireland). Samples were loaded onto a C18 trap (stainless steel trap cartridge, C18, 1 mm i.d. × 5 mm, (Part 160434, Fisher Scientific, Ireland Fisher Scientific, Dublin, Ireland)) at an approximate flow rate of 10 µL/min with 100% buffer A (LC–MS-grade water 99.9% and LC–MS grade acetonitrile with 0.1% (*v*/*v*) trifluoroacetic acid) Fisher Scientific (Fisher Scientific, Ireland Scientific, Dublin, Ireland). Each sample was loaded onto a C18 analytical column Aurora UHPLC column (25 cm × 75 μm ID, C18, 1.6 μm) (Ionopticks, Fitzroy, Australia). Separation was carried out in a linear gradient at a flow rate of 300 nL/min, with buffer B increasing from 5 to 32% during 0 to 23 min, from 32 to 95% between 23 to 24 min, and at 95% between 24 to 30 min. The mass spectrometer, Bruker timsTof Pro, was operated in positive ion mode with a capillary voltage of 1500 V, dry gas flow of 3 L/min, and a dry temperature of 180 °C. All data was acquired with the instrument operating in trapped ion mobility spectrometry (TIMS) mode. Trapped ions were selected for ms/ms using parallel accumulation serial fragmentation (PASEF). A scan range of 300–1500 *m*/*z* was performed at a rate of 10 PASEF MS/MS frames to 1 MS scan with a cycle time of 1.15 s.

### 2.8. MS Proteome Data Analysis

All raw files were processed using FragPipe (v.14) pipeline consisting of MSFragger (v.3.1.1), Philosopher (v.3.3.12), and Python (v.3.7.8). Data were searched against a fused target/decoy database generated by Philosopher and consisting of human UniProt SwissProt sequences (Homo sapiens, 11 January 2021 release), plus common contaminants. The database had 84,754 entries (including 50% decoy, 42,377 entries). MSFragger parameters were set to allow a precursor mass tolerance of plus/minus 50 ppm and a fragment tolerance of 20 ppm. Peptides were required to be fully tryptic with a maximum of two missed cleavage sites; carbamidomethylation of cysteine residues as fixed modification, and methionine oxidation and protein N-terminal acetylation as variable modifications. ID validation was performed with PeptideProphet and ProteinProphet [21] using the default “closed search” parameters.

For quantification of proteins, label-free quantification (MaxLFQ) with IonQuant was performed using default settings, including default normalisation method. Briefly, feature detection tolerance was set to 10 ppm, “Match-between-runs” (MBR) was enabled to a retention time window of 20 min and an ion mobility window of 0.05 1/K0; minimum isotope count was set to 2 by default. Default false discovery rate (FDR) options were applied at 1% across ion-level, peptide and protein. For each biological sample, two technical replicates were combined using the average of both protein abundance values. Protein group databases are listed in Supplementary Materials.

### 2.9. Quantitative Analysis

Bioinformatic analysis of the Fragpipe output files and data visualization were performed with Perseus software (v 1.6.15.0) and RStudio employing the following packages: ggplot2, ggpubr, ComplexHeatmap, and ClusterProfiler. Perseus software was used to transform LFQ values (log2), and a protein was considered quantified only if it was detected in at least four out of five biological replicates. Missing values were imputed using the MinProb function set to q = 0.01 by default. Principal component analysis (PCA) was performed in Perseus using default parameters. Scores were calculated by subtracting the mean of protein abundance values in all samples and dividing by the standard deviation. Gene Ontology analysis was performed using the ‘enrichGO’ function of the cluster Profiler R and Bioconductor package with parameters ‘pAdjustMethod = ‘FDR’; ont = ‘BP’; qvalueCutoff = 0.01; Homo sapiens (human) genome/proteome obtained from DAVID tool used as background for each GO analysis [22]. Each gene ontology dataset was independently analysed and visualised by the ggpubr package.

### 2.10. Histone PTM Analysis

A total of 30 µg of histone extracts were resuspended in 30 μL of 50 mM NH_4_HCO_3_ (pH 8), mixed with 15 μL derivatization mix (propionic anhydride and acetonitrile in a 1:3 ratio (*v*/*v*)), immediately followed by the addition of 7.5 μL ammonium hydroxide to maintain pH 8.0 [23]. The samples were incubated for 20 min at room temperature and subsequently dried in a SpeedVac. The reaction was performed twice to ensure derivatization completion. Next, samples were reconstituted in 50 mM NH_4_HCO_3_ and incubated with trypsin (enzyme:sample ratio of 1:20) overnight at room temperature. The derivatization procedure was repeated after digestion to derivatize peptide N-termini [23]. All samples were desalted prior to nanoLCMS/MS analysis using in-house-prepared C18 stage tips and stored at −80 °C until further use.

### 2.11. Histone Data Analysis

Data were analysed by using EpiProfile 2.0 [24], and a label-free strategy was selected. The peak extraction mass tolerance was set to 10 ppm. The peptide relative ratio was calculated by considering the peak area of all peptides that share the same amino acid sequence as total peptide abundance, and estimating the percentage of each individual species by dividing the peak area by the total peptide abundance.

### 2.12. NanoLCMS/MS for Histone Peptide Analysis

After drying, histone samples were resuspended in 10 μL of LCMS-grade water + 0.1% TFA and 0.5% acetic acid, and analysed by nanoLCMS/MS with a nanoLC (Dionex Ultimate 300, Fisher Scientific, Dublin, Ireland; in-house column using reverse-phase 75 μm ID × 20 cm Reprosil-Pur C18-AQ 3 μm; Dr. Maisch GmbH) coupled to an Orbitrap Q Exactive mass spectrometer (Fisher Scientific, Dublin, Ireland). Hi3 peptide standards were spiked in according to the manufacturer’s instructions and eluted together with the sample for quality control. The nanoLC pumped a flow-rate of 350 nL/min with a programmed gradient from 0.5% to 23% solvent B (A = 0.5% Acetic Acid; B = 80% acetonitrile) over 61 min, followed by a gradient from 23% to 95% solvent B in 5 min. Data were acquired using a data-independent acquisition method. Specifically, a full-scan MS spectrum (*m*/*z* 300–1100) was acquired in the Orbitrap with a resolution of 140,000 and an AGC target of 1 × 10^6^, followed by 16 MS/MS windows of 50 *m*/*z*. MS/MS was performed with a resolution of 17,500 with an AGC target of 1 × 10^6^. MS/MS was acquired using higher-energy collisional dissociation with normalized collision energy of 30.

### 2.13. Histone Peptide Pull-Down Assays

Peptide pull-down assays were performed as described previously [25] with minor modifications. Biotinylated histone H3K4 and H3K27 trimethylated (me3) peptides and relative unmodified (me0) motifs were purchased from ActiveMotif (Austria). Histone peptide pull-down assays were performed using the automated KingFisher DuoPrime purification system (Fisher Scientific, Dublin, Ireland) in a series of steps. First, 0.5 μg of each histone peptide was coupled to 0.1 mg of streptavidin-coated Dynabeads M280 in 50 μL of coupling buffer (25 mM Tris-HCl pH 8.0; 1 M NaCl; 1 mM DTT; 10% glycerol; 0.1% NP-40) at 4 °C for 2 h, mixed at low speed. After one washing step to remove the unbound peptide, samples were incubated with 0.5 mg of chromatin lysates (previously described) for 3 h at 4 °C mixed at low speed. Next, unbound proteins were removed by three washing steps.

Briefly, samples were collected with the magnetic arm of the KingFisher and transferred to be washed in 3 different deepwells containing washing buffer with detergent (20 mM HEPES [pH 7.9]; 150 mM KCl; 1 mM DTT; 0.1% NP-40; proteinase inhibitors) for 1 min at 4 °C, mixed at fast speed. The ultimate washing step excluded detergent to avoid any contamination into the LCMS/MS. For western blotting analysis peptide-bound proteins were eluted by heating the beads in 100 μL of 2 × SDS-sample buffer containing 5% β-mercaptoethanol at 95 °C for 10 min. For histone peptide pull-down mass spectrometry analysis samples were eluted in 25 ul of elution buffer (200 mM Glycine pH 2.5; 100 mM NH₄OH pH 10.0) for 10 min at 4 °C mixed at low speed.

## 3. Results

### 3.1. Expression Proteomics of Biochemically Separated Subcellular Fractions from a Model of Pluripotent Cell Differentiation

**Figure 1:**
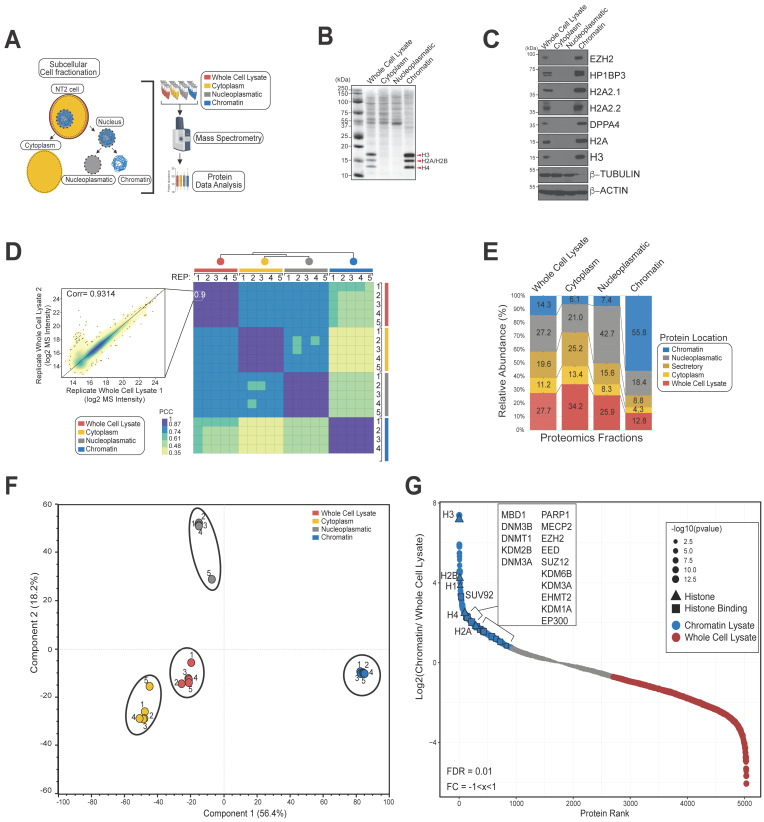
Expression proteomics of biochemically separated subcellular fractions in a cultured cell pluripotency model. (**A**) NT2 cells were separated into cytoplasmic, nucleoplasmic, and chromatin fractions and analysed by LCMS. (**B**) The fractions were separated by SDS-PAGE; prominent histone bands localizing to the chromatin fractions are arrowed. (**C**) Western blotting was used to confirm successful separation using fraction-specific marker proteins. (**D**) Pairwise correlation analysis confirmed high reproducibility of the LCMS platform for replicate analysis of each fraction (n = 5). (**E**) Relative proportions of proteins annotated to be located in the listed cellular compartments. (**F**) Experimental separation of the requisite fractions and replicate analyses were confirmed using principal component analysis. (**G**) The chromatin fraction is rich in histones and chromatin-modifying proteins (box inset). Ion intensities for >5000 proteins identified in the various cell fractions were ranked by enrichment in the chromatin fraction relative to cell lysate. Original Western blot images can be found in Supplementary Figure S1.

In order to investigate expression changes to the NT2 cell proteome over time, we first established a platform for comprehensive expression proteomics analysis. Nuclei were harvested from unstimulated cell lysates (i.e., in the pluripotent state) and divided into chromatin and nucleoplasmic fractions using biochemical methods (Figure 1A). The cytoplasmic fraction was also retained. Following trypsin digestion and sample clean up, these fractions were subjected to liquid chromatography mass spectrometry (LCMS) analysis using a timsTOF Pro 2 employing parallel accumulation serial fragmentation (PASEF). Overall, 6213 proteins were quantified among cytoplasmic, nucleoplasmic, and chromatin fractions (Supplementary Table S1).

SDS-PAGE analysis was used to visually confirm successful fractionation, with histone proteins locating to the chromatin fraction (Figure 1B). We also confirmed that the localization of key marker proteins to the expected fractions. Various nucleosomal histone molecules (H2A, H3), the heterochromatin component HP1BP3, the PRC2 complex methyltransferase EZH2, and the pluripotency marker DPPA4 all localized exclusively to the chromatin fraction. In contrast, β-tubulin located to the cytoplasm and nucleoplasm fractions, while β-actin (present throughout different compartments in mammalian cells and a component of several distinct chromatin remodelling complexes) was found in all tested fractions (Figure 1C).

We next assessed the robustness and reproducibility of our LCMS analysis platform. Each fraction was analysed using five biological replicates and correlation coefficients (Pearson) were calculated for each pair of experiments. Replicate experiments showed high reproducibility (PCC > 0.87), while as expected, comparison of protein expression profiles across different cellular fractions showed lower levels of similarity (PCC = 0.35–0.74) (Figure 1D). We counted the relative quantified proportions of proteins deemed to be located in ‘chromatin’, ‘nucleocytoplasmic’, ‘secretory’, ‘cytoplasm’, and ‘whole cell lysate’ compartments using the Human Protein Atlas Database and found that the enrichment of each cell compartment was as expected (Figure 1E). In particular, we observed an overall increase in the proportion of proteins classified as chromatin-associated protein within the NT2 chromatin sample. Principal component analysis (PCA) confirmed that data originating from distinct cell fractions were effectively resolved in both PCA dimensions, while data arising from replicate experiments projected close together (Figure 1F).

Finally, we plotted enrichment ratios based on ion intensity recorded for proteins in the chromatin fraction relative to that for all proteins detected in total cell lysate (Figure 1G). High-ranking proteins based on this analysis that showed strong enrichment (>2-fold) included histones and many chromatin-associated factors. These factors included proteins associated with DNA methylation processes (DNMT1, DNM3A, DNM3B, MECP2, MBD1), histone-modifying enzymes including acetyltransferases and deacetylases, methyltransferases and demethylases (EP300, HDAC1, EHMT2, KDM2B), Polycomb proteins (EZH2, EED, SUZ12), and other well-known chromatin-associated proteins such as PARP1. In conclusion, these experiments confirmed successful establishment of a proteomics analysis platform with high specificity for distinct subcellular fractions in the cultured NT2 cell model that was capable of reproducibly quantifying over >3000 chromatin-associated proteins.

### 3.2. Molecular Functions Associated with Distinct Subcellular Fractions

**Figure 2:**
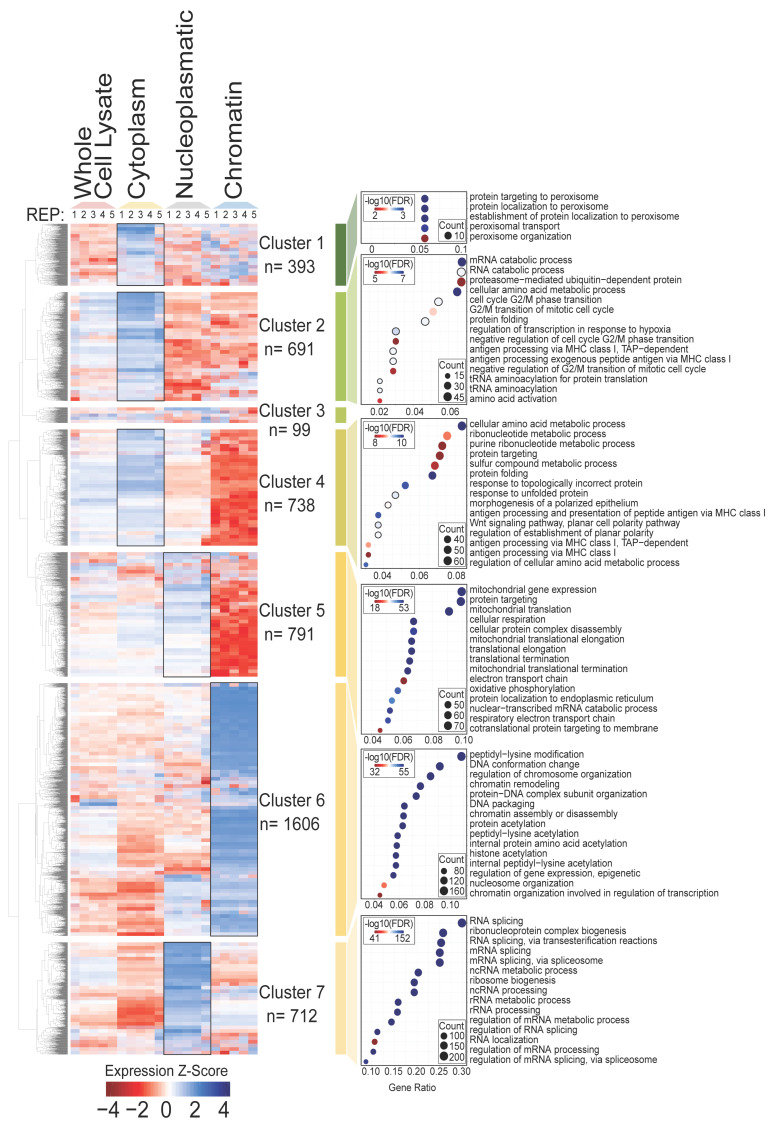
Molecular functions associated with distinct subcellular fractions. Z-scores quantifying the relative expression of proteins in the indicated cell fractions were used to classify proteins with similar expression patterns into clusters. Functional analysis showing enriched Gene Ontology categories for each cluster is shown (right).

In order to examine how protein function was distributed among separate cell compartments, z-scores quantifying the expression enrichment of each protein across the fractions were subjected to hierarchical clustering (1D), and the resulting clusters analysed using Gene Ontology classification (Figure 2). This revealed patterns of functional enrichments of proteins within distinct clusters that correspond to the known biology of the relevant cell compartments. For example, high-ranking functional enrichments in Clusters 2 and 4, mapping mainly to cytoplasmic fractions, are rich in proteins associated with processes that primarily take place in the cytoplasm, including amino acid metabolism, RNA catabolism, protein folding, and cell cycle and proteasome/ubiquitin pathways. Similarly, Cluster 1 contains proteins detected predominantly in the cytoplasm, and maps to functions important in peroxisome biology. Cluster 5, enriched in proteins from both the cytoplasm and nucleoplasm fractions, is rich in proteins involved in mitochondria biology and respiratory metabolism functions. Cluster 7, strongly enriched for nucleoplasm fraction proteins, is correspondingly rich in functions related to RNA biology that are associated with this fraction including RNA splicing, ribosome biogenesis and rRNA processing. The strongest enrichment for chromatin fraction proteins is Cluster 6, which maps to many aspects of DNA biology including chromatin and nucleosome organization and modification.

Overall, functional enrichment analysis provided independent confirmation that the various fractions used in our study were biochemically meaningful. Fruthermore Scientific, Irelandre, the most significant z-scores (indicating both increased and decreased expression relative to the pluripotent state) were consistently observed among the proteins within the chromatin fraction.

### 3.3. Chromatin Proteome Changes Induced by Differentiation

**Figure 3:**
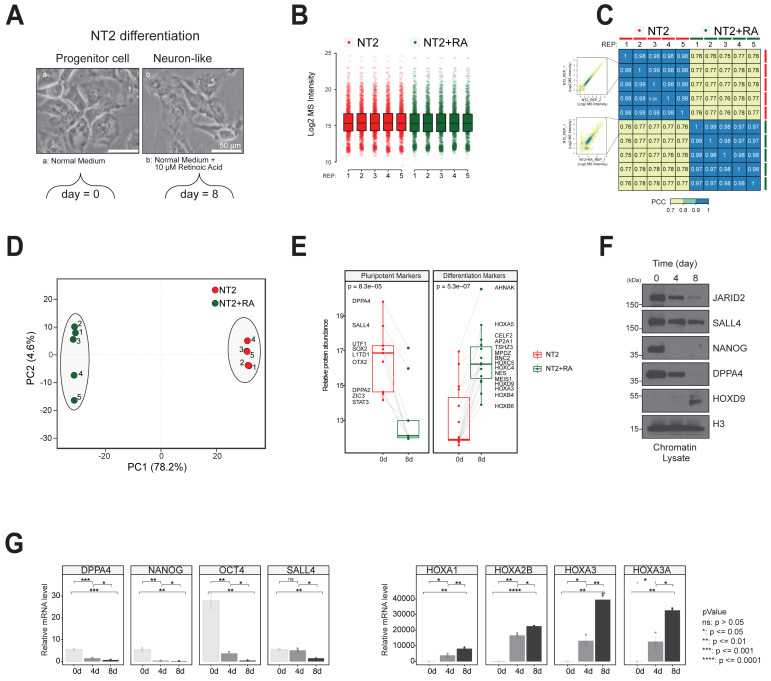
Chromatin proteome changes induced by differentiation. (**A**) Cultured pluripotent NT2 cells were induced to differentiate by treatment with retinoic acid. (**B**) Mass spectrometry signal intensities for 3740 proteins detected in the chromatin fraction were distributed evenly in both undifferentiated and differentiated cell states. (**C**) Correlation analysis of technical replicates (n = 5) for both cell states confirms strong experimental reproducibility. The insets show examples of the distribution of two technical replicates of the pre-differentiation cells (top) and two samples drawn from the pre- and post-differentiation states (bottom). (**D**) Cell state and technical replicate experiments also project onto closely clustered groups using Principal Component Analysis. (**E**) Proteins designated ‘Pluripotency Markers’ showed decreased expression upon differentiation, while proteins designated ‘Differentiation Markers’ were increased. (**F**) Western blotting confirmation of selected protein expression changes. (**G**) RT-PCR confirmation of selected mRNA expression changes. Original Western blot images can be found in Supplementary Figure S1.

We next addressed how the NT2 cell proteome evolved and responded to the major phenotype changes involved in differentiation to neuronal cells from the pluripotent state. We used retinoic acid treatment to induce differentiation, harvesting the chromatin fraction at Day 0 (pre-induction) and Day 8 (post-induction) (Figure 3A). Five biological replicates were analysed for both cell states, with similar protein abundance distributions measured across 3740 identified proteins (Figure 3B) (Supplementary Table S2). The LCMS platform showed high measurement reproducibility. Replicate experiments within the same cell state displayed Spearman’s Rank Correlation Coefficients of 0.97–0.98, while those recorded between states ranged 0.75–0.78 (Figure 3C). This gave confidence in the biological relevance of proteins found to significantly alter expression after treatment with retinoic acid. PCA analysis of these data also found a wide separation of the cell state experimental data across Dimension 1 (78.2%), alongside close clustering of replicate samples across Dimension 2 (4.6%) (Figure 3D). This confirms that profound proteome level changes occur in NT2 cells during the differentiation process. The strong separation of data further increases confidence that proteins deemed to change across both cell states are biologically significant.

As an additional test of the biological relevance of our data, the mass spectrometry ion signals for proteins classified as pluripotency or differentiation markers were compared (Figure 3E). The former were consistently found to decrease and the latter to increase following differentiation. Examples of pluripotency factors found to have decreased expression include SOX2, UTF1, LITD1, and ZIC2. Similarly, the expression levels of the epigenetic regulators DPPA2 and DPPA4, involved in opposing de novo DNA methylation [26], were decreased after differentiation. Among the differentiation markers upregulated following retinoic acid treatment, members of the HOX family of evolutionarily conserved developmental transcription factors were observed, including HOXA3, HOXA5, HOXB4, HOXB6, HOXC4, HOXC5, and HOXD9 [27]. Additionally, many proteins associated with neuronal development increased in expression during differentiation. These included NESTIN, AHNAK, TSHZ3, MPDZ, and CELF2, an RNA-binding protein recently linked to neuronal stem cell fate determination [28].

We confirmed several of these observations using western blotting (Figure 3F). DPPA4, SALL4, and JARID2 replicated the changes we observed for the pluripotency factor NANOG. In contrast, levels of HOXD9 were confirmed to increase during differentiation. Finally, we tested these observed protein-level expression changes at the mRNA level using RT-PCR. The same general patterns were observed following differentiation, with mRNA levels for the pluripotency markers NANOG, OCT4, DPPA4, and SALL4 all decreasing, while levels of the differentiation markers HOXA1, HOXA2B, HOXA3, and HOXA3A mRNAs were observed to increase (Figure 3G).

Overall, this work demonstrated the relevance of our experimental model for study of the differentiation of cultured pluripotent cells while highlighting the particular significance of chromatin-associated proteins to that process.

### 3.4. The NT2 Cell Differentiation Programme Centres on Processes Involving Chromatin Modification Pathways

**Figure 4:**
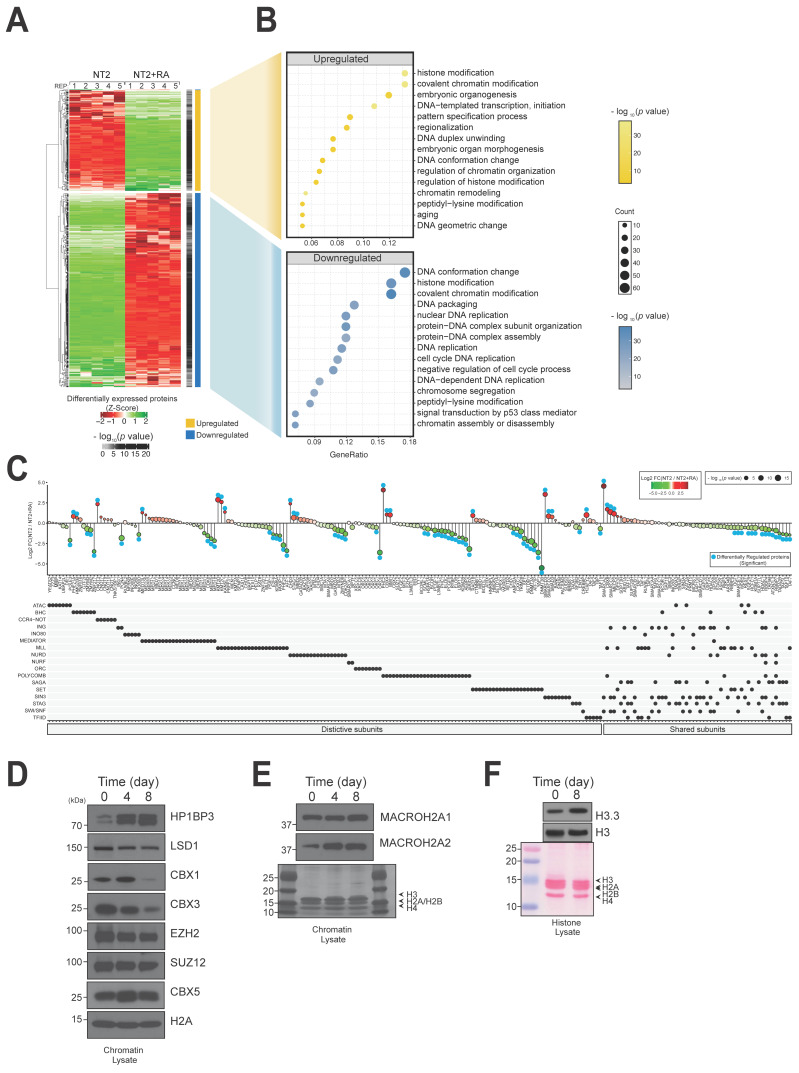
NT2 cell differentiation programme centres on processes involving chromatin modification pathways. (**A**) Heat map plotting z-scores of 166 up-regulated and 320 down-regulated proteins following treatment with retinoic acid. (**B**) Differentially expressed proteins mapped to functional pathways governing gene expression regulatory activities. (**C**) Protein expression changes for components of chromatin-modifying complexes. (**D**) Western blotting confirmed protein expression changes observed in the proteomics experiment for histone proteins and chromatin-modifying enzymes. (**E**) Western blotting confirmed protein expression changes observed in the proteomics experiment for macroH2A1 and macroH2A2. (**F**) Western blotting confirmed protein expression changes observed in the proteomics experiment for histone H3 and H3.3. Original Western blot images can be found in Supplementary Figure S1.

From 1384 chromatin associated proteins, approximately 35% were found to be differentially regulated (Supplementary Table S2). In particular, 166 were found to be up-regulated in the differentiating cells, and 320 down-regulated (0.01 *t*-test; *p* < 0.01) (Figure 4A). Both classes mapped to functional pathways linked to chromatin modification and the regulation of aspects of DNA structure relevant to gene expression, replication, and cell cycle processes (Figure 4B). While functions associated with chromatin proteins are expected in a chromatin-associated fraction, the particular focus on chromatin biology is consistent with a coordinated programme of gene expression change centred around the control of access to DNA in order to regulate the activation and repression of distinct sets of genes.

Developmental epigenetic programmes are commonly controlled by heteropolymer macromolecular complexes such as the Polycomb Repressor Complex 2 (PRC2) that induces repression at targeted genome regions by methylating specific histone H3 lysine tail residues (H3K27me3). In addition to protein-modifying activities (addition and removal of acetyl, methyl groups, etc.), Polycomb and other such complexes regulate access to the underlying DNA by the transcriptional machinery through the remodelling of nucleosome structure. We plotted expression changes for the individual protein components of these complexes in order to visualize patterns of behaviour during differentiation (Figure 4C). In most cases, the core complex components (i.e., the component proteins described in in vitro reconstitution experiments or those resolved in structural studies) were coordinately expressed in either cell state, while accessory/regulatory proteins showed stronger expression changes. For example, the core PRC1 complex proteins (BCOR/BCORL1) show increased expression in differentiated cells, as do core subunits of the SET (SETD1A, SETD2A, WDR5, ASH2L, RBBP5) and Origin Recognition (ORC1, ORC2, ORC3, ORC4, ORC5, ORC6) complexes. Non-core accessory proteins linked to these complexes (e.g., CBX4, PHC2 for PRC1 complex; AEBP, JARID2 for PRC2 complex; XRCC5, XRCC6 for origin recognition complex) show stronger congruent changes relative to other complex subunits, or in some cases, incongruous changes (e.g., DPY30 for SET complex). This is consistent with the coordinated assembly and constituent expression of chromatin-modifying machinery during differentiation, subject to control by accessory subunits, similar to the ‘just-in-time’ hypothesis originally proposed for yeast cell cycle complexes [29]. Other complexes show similar patterns but in the opposite direction. For example, members of the CNOT deadenylation complex (CNOT1, CNOT3 CNOT7, CNOT9, CNOT10) show elevated expression in undifferentiated cells, but a newly identified subunit from human cells (TNKS1BP1) is elevated in differentiated cells [30]. Other complexes, such as the mediator complex, which is capable of integrating diverse regulatory inputs through its association with RNA polymerase II transcription, show a mixed response.

We validated our analysis on histone and histone-associated proteins found to increase upon differentiation (histone H2A variants “H2A2.1” and “H2A2.2”, HP1BP3), heterochromatin proteins that decrease upon differentiation (CBX1, CBX3L), and histone modification enzymes that are expressed at relatively constant levels in both cell states (CBX5, EZH2, SUZ12) (Figure 4D). Replacement of histone proteins with related variants on selected nucleosomes is another epigenetic mechanism employed during widespread gene expression changes. We also confirmed two interesting findings from the chromatin expression proteomics experiments on altered levels of histone variants in the pluripotent and differentiated cell states by western blotting (Figure 4E). We found that levels of macroH2A, which contains a large C-terminal extension relative to canonical H2A, increased during differentiation (Figure 4E). This variant may protect extranucleosomal DNA within the chromatin environment and contribute to more stable histone octamers and is speculated to impede reprogramming functions [31]. Similarly, we found that levels of the histone H3 variant H3.3, associated with high-turnover dynamic chromatin domains [32], increased strongly during the differentiation of NT2 cells relative to the canonical H3.1 form. Western blotting confirmed that global levels of histone H3.3 were increased during differentiation (Figure 4F). Overall, expression proteomics analysis focused on the chromatin fraction of differentiating NT2 cells reveals regulatory changes that centre on components of chromatin modification complexes, as well as specific histone variants.

### 3.5. Analysis of Histone Post-Translational Modification Changes during Differentiation

**Figure 5:**
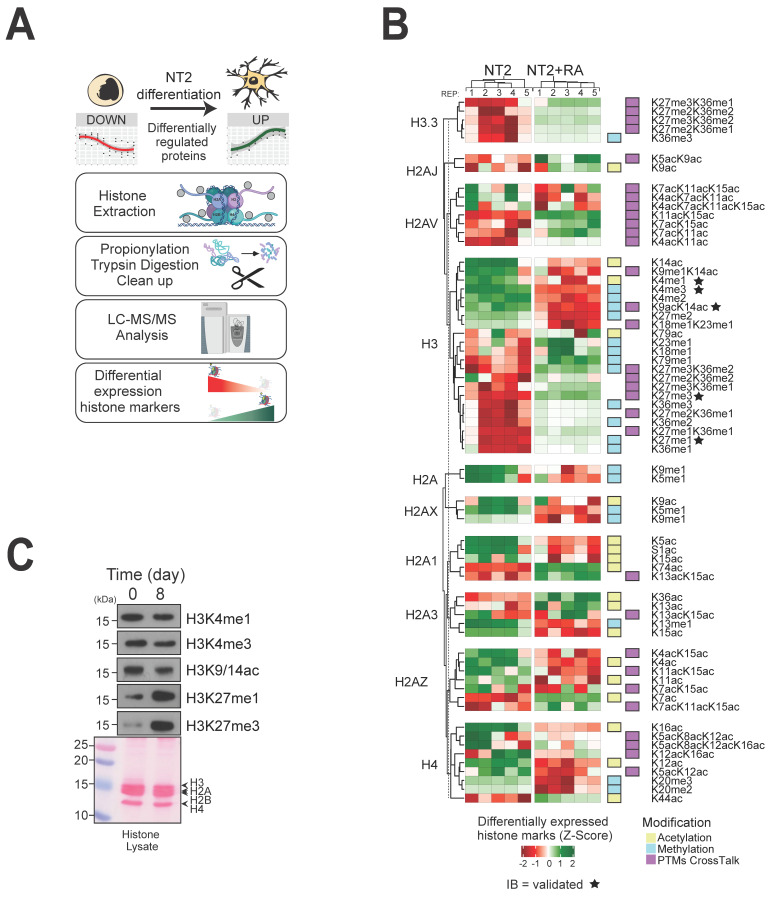
Global histone post-translational modification levels are significantly altered during differentiation. (**A**) Relative levels of histone modifications in the two NT2 cell states were determined using a propionylation-based chemical proteomics approach. (**B**) Heat map showing relative levels of modifications before and after treatment with retinoic acid (n = 5). The green/red fold-change scale is based on the extracted ion signal recorded for the modification. (**C**) Altered global levels of histone H3 lysine modifications during the differentiation cycle were confirmed using western blotting. Original Western blot images can be found in Supplementary Figure S1.

Since we found that many histone modification enzymes and protein complexes were regulated during the differentiation process, we next aimed to quantify any changes to global levels of the modification states of their histone substrates (Figure 5A). We again used mass spectrometry, employing a chemical derivatization procedure to compare the relative abundance of histone acetylation and methylation modifications before and after treatment with retinoic acid [33]. Overall, 140 distinct modification sites were measured across twelve histone proteoforms (Supplementary Table S3). Relative ion signals for each modification were plotted on a heatmap to highlight histone marks showing 67 significant modification site changes during NT2 cell differentiation (Figure 5B).

This analysis revealed complex patterns of histone mark change; for example, global levels of two acetylation modifications of histone H2A that are associated with active transcription start sites (H2AK5ac, H2AK9ac) were strongly downregulated during differentiation. Other histone PTMs associated with active transcription on histone H4 (H4K12ac, H4K16ac) were similarly downregulated. Acetylation and methylation changes observed on histone H3 varied depending on the specific PTM (or combination of PTMs). A general downregulation of histone marks associated with active transcription (H3K14ac, H3K79ac, H3K4me1/me2/me3) and an upregulation of marks associated with gene repression (H3K27me3) were observed. Other histone H3 PTMs that likely perform buffering functions were upregulated upon differentiation; for example, H3K36me2 may delineate active and repressed chromatin domains [20]. We validated several of these observations using histone PTM-specific antibodies: downregulation of H3K4me1, H3K4me3, and H3K9ac-K14ac and upregulation of H3K27me1 and H3K27me3 (Figure 5C).

In summary, these data provide a valuable overview of global histone PTM changes recorded over a cultured cell differentiation programme and reinforce the key role that histone-modifying enzymes and enzyme complexes play in these processes.

### 3.6. Histone Post-Translational Marks Associate with Distinct Protein Subsets during the Pluripotent and Differentiated States

**Figure 6:**
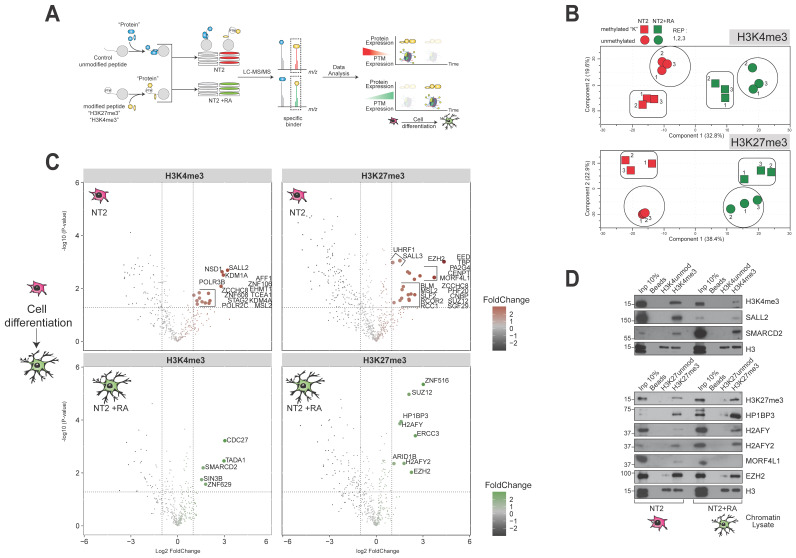
Histone post-translational marks associate with distinct protein subsets during the pluripotent and differentiated states. (**A**) Overview of the histone tail peptide pulldown experiment. (**B**) Principal Component Analysis of peptide pulldown experiments with histone H3K4, H3K4me3, H3K27, and H3K27me3 (n = 3). (**C**) Volcano plots projecting fold-change observed against significance (*t*-test) for all four experiments. (**D**) Western blot analysis of selected results from peptide-pulldown experiments. Original Western blot images can be found in Supplementary Figure S1.

Modifications of histone molecules, including acetylation and methylation, are dynamically deposited (“writing”) and removed (“erasing”) during differentiation, and the modification status is continually “read” by binding modules within multifunctional proteins or protein complexes. For this reason, we next sought to identify the cohort of proteins that recognize key histone modifications (Figure 6A). We used a peptide pulldown strategy to investigate protein binding to histone epigenetic marks, since many of the proteins and pathways that were found to display significant expression changes in response to differentiation are involved in the addition or removal of histone modifications (Supplementary Table S4). Matched pairs of biotinylated peptides (one unmodified, one trimethylated) were added to nuclear lysates from differentiated NT2 cells in parallel, affinity-purified using streptavidin beads, and the resulting captured proteins were analysed using LCMS [25].

Experiments targeting the H3K4me3 and H3K27me3 modifications (observed to decrease and increase global levels following differentiation, respectively; Figure 5B,C) were carried out. The key finding was that histone tail peptides associate with quite distinct sets of chromatin proteins in the pluripotent and differentiated states. This is highlighted by Principal Component Analysis of the results, which found that cell state differences were responsible for the majority of changes observed in the histone mark-binding proteome (32.8–38.4%). Differences assigned to the methylated state (i.e., naked versus methylated synthetic peptides) were actually of lower magnitude (19.6–22.9%), while experimental replicates showed strong concordance, confirming the reliability of the data (Figure 6B). The individual proteins were projected onto volcano plots mapping enrichment in methylated peptide pulldowns relative to unmethylated sister peptides (*x*-axis) against significance (*t*-test, *p*-value; *y*-axis) (Figure 6C). Notably, the complement of H3K4me3- and H3K27me3-binding proteins changes over the differentiation cycle. Transcription factors associated with the pluripotent state, such as SALL2 and NSD1 associated with the H3K4me3 peptide in undifferentiated chromatin fractions, while components of more general chromatin-modifying machinery such as SMARCD2 and SIN3B were enriched in differentiated cells. Other zinc finger proteins and potential transcription factors like ZNF608 and ZNF629 associated with H3K4me3 in alternative cell states but remain poorly studied. For H3K27me3, a protein complement that included the core enzyme complex components responsible for catalyzing H3K27me3 production, EZH2 and SUZ12, was found across both cell states, while other proteins including transcription factors and chromatin-modifying complex components such as SALL3, UHRF1, MORF4L1, ARID1B and ZNF516 were specifically associated with either cell state. These observations were independently confirmed for selective examples from three experiments (biological replicates) using peptide pulldown-western blotting (Figure 6D).

### 3.7. Histone Variant and Histone Proteolysis Changes Induced by Differentiation

**Figure 7:**
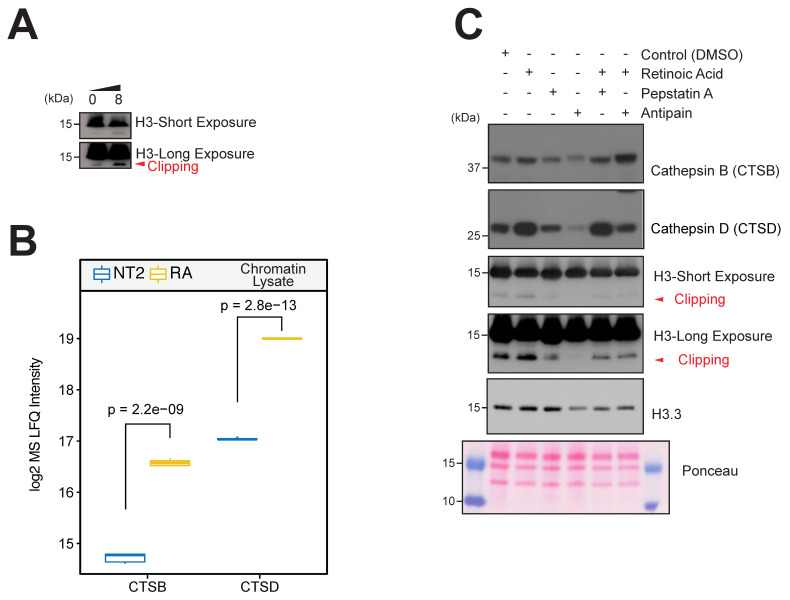
Histone variant and histone proteolysis changes induced by a differentiation programme. (**A**) Increased histone H3 amino-terminal tail clipping during NT2 cell differentiation was detected by western blotting. (**B**) Analysis of mass spectrometry ion signals confirms increased levels of cathepsin B (CTSB) and cathepsin D (CTSD) following differentiation. (**C**) Western blot analysis of full- length and clipped histone H3 levels in the presence or absence of the protease inhibitors pepstatin A or antipain, in undifferentiated or differentiated (+RA) cells. Original Western blot images can be found in Supplementary Figure S1.

In earlier experiments, we noticed increased antibody-reactive staining below the full-length histone H3 band when comparing levels of variant histones before and after differentiation. We confirmed these observations by western blot (Figure 7A). These products arise following the proteolytic removal of histone tail fragments and are proposed to provide a mechanism for rapid epigenetic regulation [34]. Several distinct proteases and protease classes have been implicated in histone clipping. For example, cathepsin family proteases have been shown to catalyse the clipping of histone H3 in mES cells [35]. We confirmed that the levels of cathepsins B and D were increased in the differentiated state using MS ion signals (Figure 7B). We treated cultured NT2 cells with the broad-specificity protease inhibitors pepstatin A and antipain before and after differentiation and probed for histone H3 clipping. This experiment found that differentiation-associated histone H3 clipping was indeed reduced in both cell states following the addition of either inhibitor (Figure 7C). We also determined the relative abundance of cathepsins B and D under the same conditions and found that the abundance of both proteases was responsive to the presence of the inhibitor. In other words, the presence of protease inhibitor led to decreased cathepsin expression in uninduced cells, or increased expression in differentiated cells. Overall, these experiments implicate a role for H3 clipping in cultured NT2 cell differentiation and Fruthermore Scientific, Irelandre suggest the involvement of proteases cathepsin B and L in at least one upstream step of the histone H3 clipping process.

## 4. Discussion

In this study, we attempted a near-comprehensive analysis of proteome-wide changes induced by the differentiation of a widely used differentiation cell model (NT2 cells). Recent notable proteomics studies have followed the response to differentiation in distinct cell contexts using similarly powerful proteomics approaches [36,37,38,39]. We began with a characterization of the major cell fractions (cytosol, nucleoplasm, chromatin) and subsequently focused on changes in the chromatin fraction when it became evident that widespread changes among chromatin-associated proteins occur upon differentiation. Proteins from many heterooligomeric complexes linked to chromatin biology were found to change expression levels during differentiation, particularly those linked to chromatin remodelling and the addition and removal of histone PTMs.

Since many of the changes that we and others observe during differentiation map to enzymes and enzyme complexes that alter the modification status of histone proteins, a major determinant of epigenetic state, the next logical steps were to investigate (a) changes to the relative histone PTM abundance between the pluripotent and differentiated states and (b) changes in binding to modified histones by reader moieties. This revealed global changes to histone PTM patterns that likely reflect the epigenetic regulation of coordinated gene expression programmes as the cell moves from the pluripotent to the differentiated state. Surprisingly, we also found that the complement of proteins that physically associate with synthetic modified and unmodified histone tail peptides varies quite significantly across the differentiation cycle.

The H3K4me3 histone modification is associated with the promoters of actively transcribed genes. This may explain our finding of peptide binding to RNA polymerase subunits (POLR2C, POLR3B) and transcription elongation factors (TCEA1) in undifferentiated NT2 cells. It may also explain the pulldown of AFF1, a member of the super elongation complex that regulates the transition from transcription initiation to elongation, which has affinity for the combined H3K4me3/H3K9Ac marks [40]. The SALL2 transcription factor is notable for its role in neurogenesis and neurodifferentiation, as well as in many cancers [41]. The SALL2 gene is regulated positively and negatively by the upstream transcription factors AP4 and WT1, respectively [42], while, in turn, SALL2 can regulate downstream targets coordinating cell cycle progression such as CDKN2A [43]. SALL2 contains multiple zinc finger domains and can interact with the NuRD chromatin remodelling complex via a 12-amino acid motif in the N-terminal region [44].

In corresponding H3K4me3 peptide pulldowns from differentiated NT2 chromatin, proteins associated with the SIN3B/HDAC corepressor complex, the SWI/SNF chromatin remodelling complex, and the TADA1-containing SAGA-like HAT complex were detected. We also detected CDC27, a core component of the anaphase-promoting complex (APC)/cyclosome. Interestingly, SIN3B has been shown to be linked to the DREAM complex, a transcription complex that regulates hundreds of cell cycle genes, linked to the APC during the G1/S phase of cell cycle progression [45]. Fruthermore Scientific, Irelandre, the SAGA complex has also been functionally linked to the APC in yeast [46]. Our observations, therefore, raise the possibility that a nucleosome-associated physical link may underlie the functional connections between the H3K4me3 epigenetic mark and cell cycle pathways mediated by the anaphase-promoting complex.

The H3K9me3 histone mark is linked to heterochromatin featuring condensed DNA and the repression of gene expression. We found that multiple proteins associated with heterochromatin, including HP1 (CBX1), the H3K9-specific methyltransferases SUV39H1, SUV39H1, and SETDB1, as well as the corresponding demethylases KDM1A and KDM3A, are pulled down by synthetic H3K9me3 peptides from NT2 cells. KDM1A, a JmjC lysine demethylase (alias LSD1), is also associated with protein complexes linked to the reprogramming of gene expression, including NuRD, CoREST, and RCOR2.

KDM1A displays activity toward the histone marks H3K4me1/me2 and H3K9me1/me2. Another JmjC lysine demethylase, KDM4A, is primarily specific for H3K9me3 and shows a phenotype associated with cellular proliferation and chromatin compaction [47,48]. KDM1A is post-translationally regulated by the E3 ubiquitin ligase JADE1, also detected in our pulldowns, to control the differentiation of ESCs toward a neural phenotype [49]. The chromobox protein CBX1 binds trimethylated H3K9 residues to recruit HP1 proteins, thereby linking it with heterochromatin control. CBX1 was recently shown to mediate tissue-specific repression via the H3K27me3 mark, potentially in a complex with PurB and Sp3 [50]. Both Sp3 and the related transcription factor Sp4 were detected in our H3K9me3 peptide pulldowns. Another Polycomb protein with affinity for methylated proteins (including non-histone proteins) linked to control of the pluripotent state, L3MBTL2, was also detected. Interestingly, L3MBTL2 was found to control the degradation of methylated forms of the DNA methyltransferase DNMT1, an effect counteracted by the KDM1A demethylase [51]. Fruthermore Scientific, Irelandre, both L3MBTL2 and DNMT1 co-purified in another proteomics study linking them to the repression of the Notch signalling pathway genes [52].

Interactions between molecular assemblies with distinct binding and enzymatic activities may be the general crosstalk mechanism by which a protein with direct affinity for one histone tail PTM may engage with another. For example, we observed that several lysine demethylases preferentially pulled down by the H3K4me3 peptide from undifferentiated NT2 cells show substrate preferences for other (non-H3K4) histone marks: KDM1A favours H3K4me1/2 and H3K9me1/2 substrates, while KDM4A favours H3K4me3 and H3K9me3. Both proteins may, therefore, be recruited to target loci through indirect association and crosstalk among these histone modifications. In another example, KDM4A is recruited to H3K4me3-rich loci by KDM5A during DNA amplification events in tumours [53], with similar events occurring during maternal epigenetic inheritance in oocytes [54]. The histone methyltransferase NSD1 also exemplifies this type of crosstalk scenario. The NSD1 protein was found to bind H3K4me3 in undifferentiated NT2 cells yet favours lysine 36 of histone H3 as a substrate, where it mainly produces H3K36me2. However, NSD1 binds the EZH2 and SUZ12 components of the PRC2 chromatin repressor complex that produces H3K27me2/3, and forms part of a network defining the boundaries between H3K7me2 and H3K7me3 domains in embryonic stem cells [20]. Loss of NSD1 was recently shown to increase levels of H3K27ac in active enhancers by preventing the recruitment of HDAC1 [55]. While deconvoluting the extended interactions of histone marks and the corresponding binding proteins will be challenging, a theme is emerging whereby epigenetic domains are demarcated by the recruitment of multiple enzymes and/or enzyme complexes engaged in aligned or opposed chromatin-modifying activity.

Interestingly, when we compared our data with two other manuscripts using similar peptide pulldown approaches, the overlap in identified proteins was poor. Vermeulen and co-workers [25] studied several trimethylated histone tail peptides (H3K4me3, H3K9me3, H3K20me3, H3K27me3, H3K36me3) from cultured HeLaS3 cells, while Kunowska [56] focused on H3K4me3 (as well as H3S10ph, and combinations thereof) in MPC11 myeloma cells. The lack of mutual overlap between these two studies and ours suggests that the population of protein molecules that bind particular histone marks is highly context-dependent. Additionally, one would expect to observe differences in processes as tightly regulated as the control of gene expression during differentiation. The distinct sets of histone mark- binding proteins that we identified under identical experimental conditions in undifferentiated (pluripotent) and differentiated cells, further support this point.

One note of caution to consider when evaluating experiments employing histone modification-specific antibodies (including ChIPseq and related nucleic acid-based experiments) is the specificity of these reagents. While high mass resolution tandem mass spectrometry can, in many cases conclusively identify different structural isomers, the absolute specificity of antibodies can be difficult to confirm. Overall, however, these experiments capture the rich histone PTM binding potential of differentiating cells (while acknowledging that the peptide affinity format is a highly simplified version of the complete molecular context of nucleosomes within a living cell).

In conclusion, this work expands our knowledge by providing three important proteome-level datasets that describe the progression of a cultured cell model from the pluripotent to the differentiated state: global expression changes over the differentiation cycle, global histone PTM changes over the differentiation cycle, and the complement of proteins associating with H3K4me3 and H3K27me3 peptides over the cycle.

## Data Availability

All data is available in the online Supplementary Materials, or on request to the Corresponding Authors.

## Supplementary Materials

The following supporting information can be downloaded at: https://www.mdpi.com/article/10.3390/biom14070747/s1: Table S1. Expression proteomics; Table S2. Chromatin proteome changes induced by differentiation; Table S3. Global histone post-translational modifications; Table S4. Histone peptide PTM pulldowns; Table S5. Reagents used in the study; Figure S1. Original western blots.
