# Supplementary figures and images for: Expression Proteomics and Histone Analysis Reveal Extensive Chromatin Network Changes and a Role for Histone Tail Trimming during Cellular Differentiation
Source: Biomolecules. 2024 Jun 24;14(7):747. doi: 10.3390/biom14070747 (PMC11274982; doi:10.3390/biom14070747)

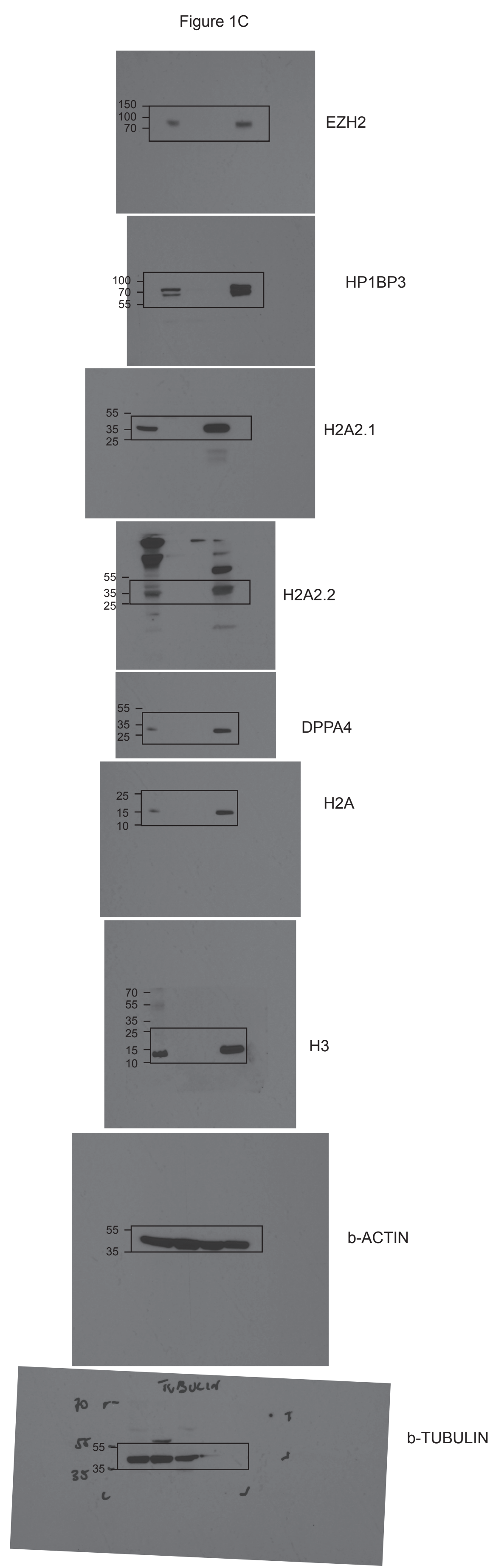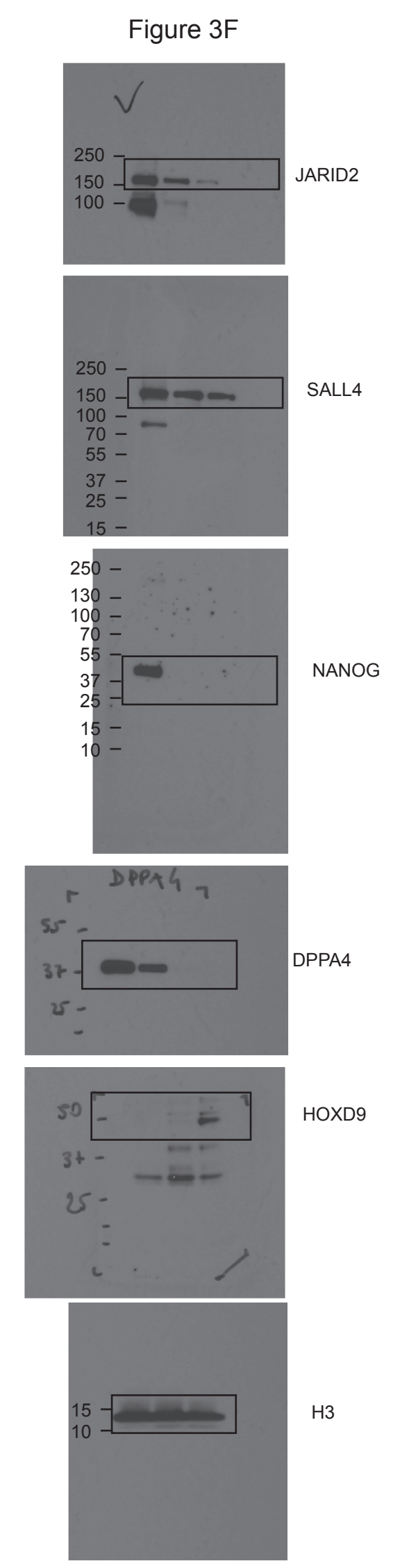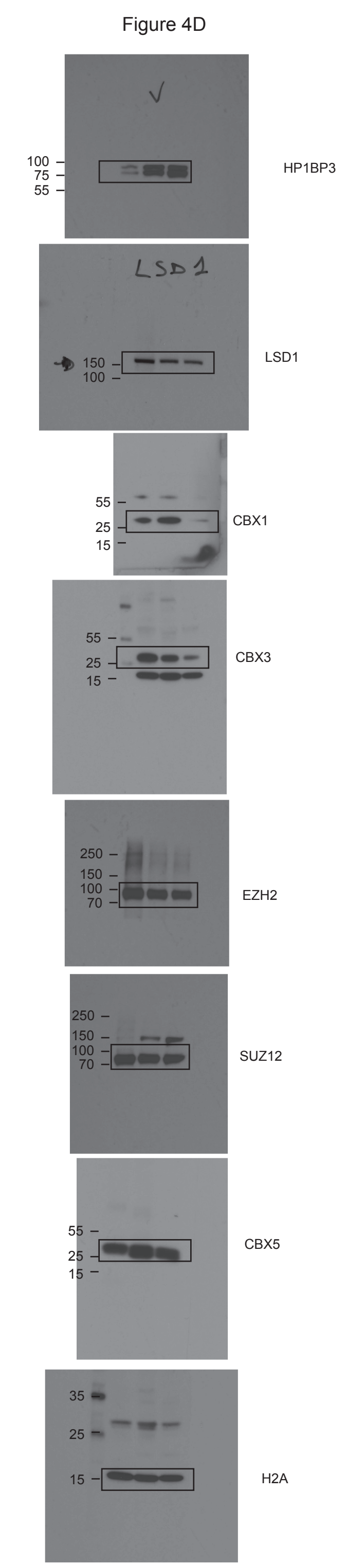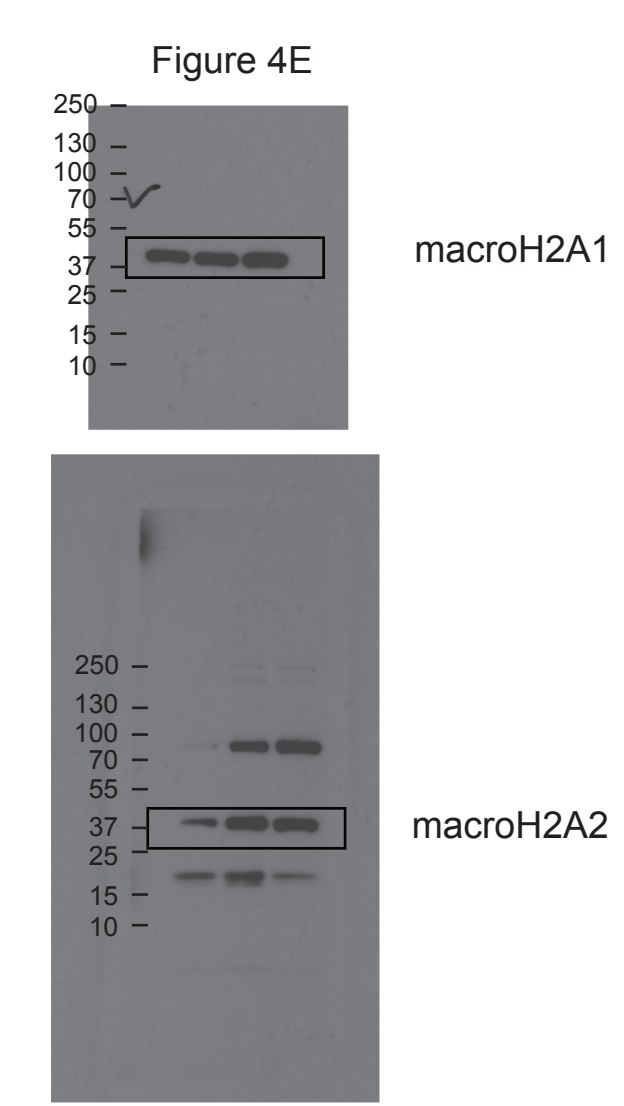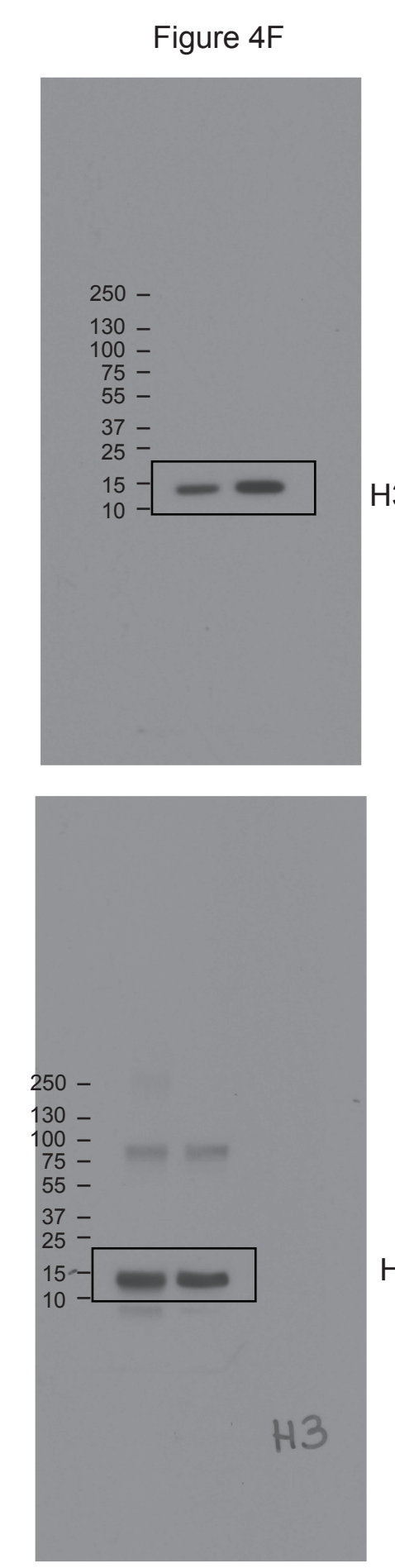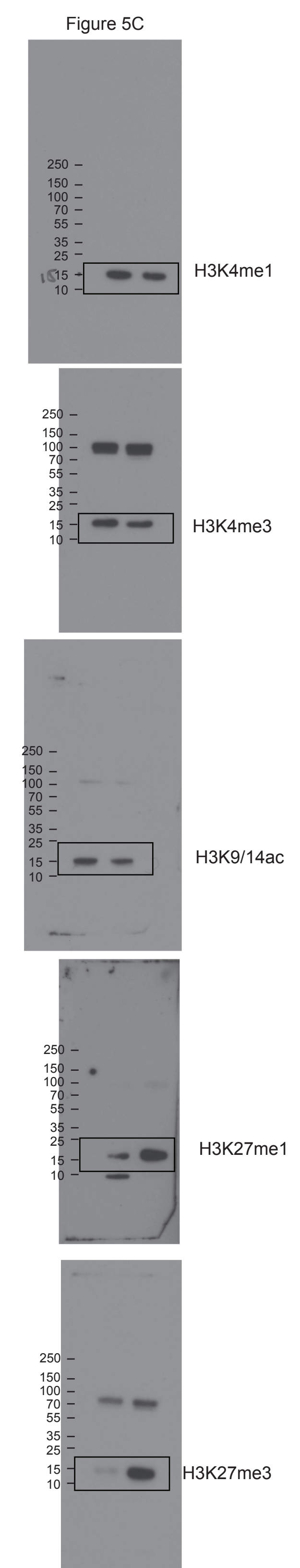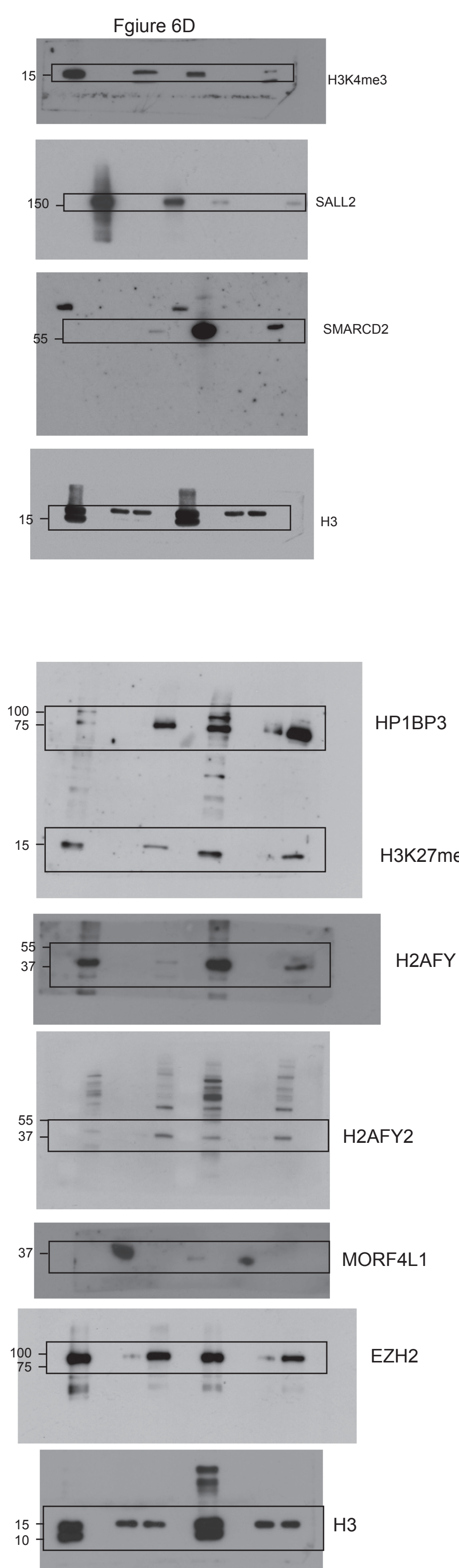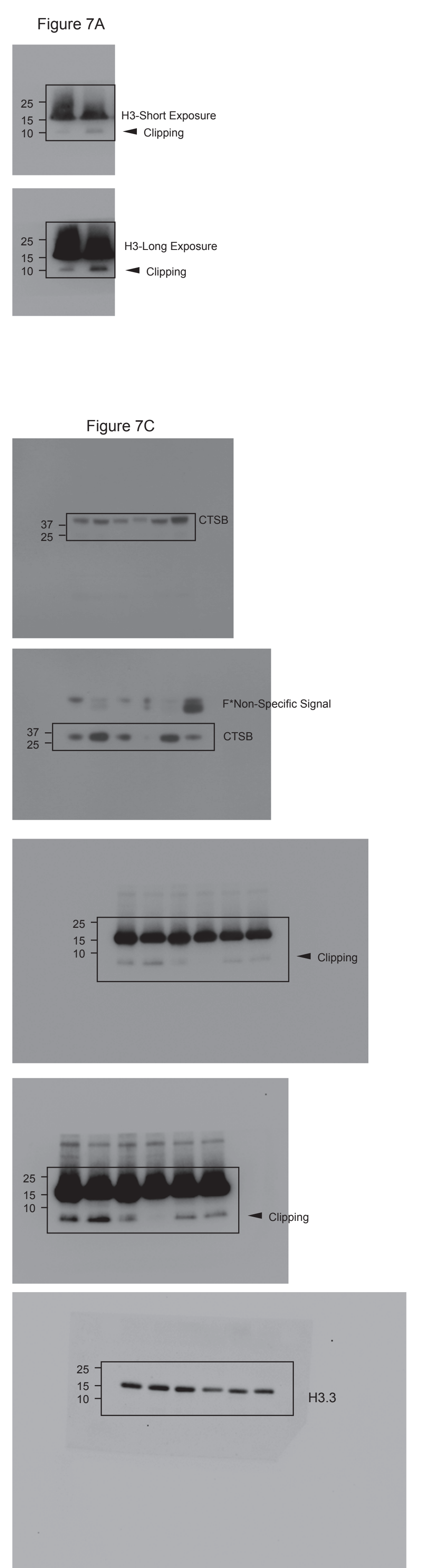

Supplement: Supplementary file 1 [file biomolecules-14-00747-s001.zip › Figure S1 Original western blots.pdf]
